# Out-of-pocket healthcare expenditures of Turkish households living with rare diseases

**DOI:** 10.3389/fpubh.2023.1051851

**Published:** 2023-03-03

**Authors:** Güvenç Koçkaya, Gülpembe Oguzhan, Selin Ökçün, Mustafa Kurnaz

**Affiliations:** ^1^ECONiX Research, Analysis and Consultancy Inc., Samsun, Türkiye; ^2^Department of Healthcare Management, Tarsus University, Mersin, Türkiye

**Keywords:** health expenditure, rare disease, Turkey, household, out-of-pocket

## Abstract

**Introduction:**

This study aims to determine the out-of-pocket health expenditures of households in Turkey where individuals with rare diseases are residing.

**Methods:**

The research population consisted registered members of associations who are members of the Rare Diseases Network. In addition to the general analysis including all participants, expenditures based on characteristics of disease holders were also calculated.

**Results:**

A total of 439 participants were included in the analysis. We determined that special nutrition was the highest expenditure group and emergency departments were the lowest expenditure group. When all the participants were evaluated, the average cost of rare diseases was found to be Ł22,743 (€2,877). A significant relationship was found between income status and out-of-pocket health expenditures (*p* = 0.012).

**Discussion:**

Policy makers should consider inclusion of special nutritional products and medical/non-medical devices used in treatment of rare diseases within the scope of reimbursement and the development of orphan drug legislation as the first actions to be taken.

## Introduction

In Turkey, diseases with a prevalence of roughly 0.1–9% for every 100,000 people are deemed rare and this rate is 50 times less common than that of the European Union and the United States. In this context, we can also say that fewer diseases are considered “rare diseases” in Turkey than in the European Union (1, 2). It is estimated that approximately 5–7 million people in Turkey have rare diseases (3).

Rare diseases cause many expenditures related to medication, healthcare use, access to treatment, special medical and nutritional needs, etc. They have indirect effects such as loss of productivity and workforce, and inability to find a job (4). The loss of income caused by inability of individuals with rare diseases to participate in the workforce and the fact that one of the family members quits his or her job to look after that individual brings a socioeconomic burden with it (5).

Predictive and preventive health services are one of the main aims of health policy in Turkey. Since Turkey possesses a social state perceptive, it is obliged to protect the health right of all its citizens. In this context and in line with the Health Transformation Program established in 2003, steps have been taken to ensure that everyone has equal and equitable access to health services (6). Access to health services in Turkey has considerably increased with the Health Transformation Program. The total number of applications made to physicians which was 208,966,049 in 2002 increased to 600,261,131 in 2020. The number of applications made to a physician per capita increased to 7.2 in 2020 while it was 3.1 in 2002. The annual number of applications was compared among Organization for Economic Co-operation and Development (OECD) countries and it was found to be 7.2 in Turkey ranking the 7th, though it was announced as 6.6 in OECD countries. According to the statistics, the total number of applications increased from 91,949 to 171,259 within the years 2002 to 2020, respectively, and the total number of physicians per 100,000 individuals increased from 138 to 205. In addition, although the total number of applications per year is higher than the ones in OECD countries, the total number of physicians per 100,000 people is higher in OECD countries than it is in Turkey (356 vs. 205) (7).

General Health Insurance (GHI) is an insurance model in which citizens contribute in direct proportion to their solvency and use the services according to their needs. It is aimed to ensure that the entire population is covered by compulsory health insurance and that everyone has equal access to health services through GHI (8). As of April 2021, 99% of Turkey's population is covered by GHI. GHI covers the health services such as emergency, intensive care and cancer treatment (9). GHI was one of the major steps taken for the whole population to have access to health services.

Health services that are not covered or are partially covered by GHI are covered through out-of-pocket health expenditures or private health insurance. Out-of-pocket health expenditures include payments made by individuals or households for the health services they receive (27). They also include payments made by individuals for the healthcare they receive that are not reimbursed by any person or institution (10). Out-of-pocket healthcare expenditures made by people with rare diseases constitute a huge burden for individuals and families (11). There is no special article for rare diseases in the GHI regarding insurance coverage and people with rare diseases and common diseases are treated the same. Although common diseases and rare diseases are equally treated, the low number of physicians dealing with rare diseases and the high costs of the treatments create a serious problem for the access of patients with rare diseases to treatments.

Rare diseases can have quite different clinical features than common diseases. For this reason, the health policies applied in the treatment of rare diseases should be different, but this is not the case in Turkey. Some workshops and reports have been conducted by the Ministry of Health on rare diseases and orphan drugs in Turkey, but no national action plan has been prepared and there is no regulation on rare diseases and orphan drugs. The progressive, life-threatening and multidimensional nature of rare diseases requires the development of an effective health policy (12). The fact that policy makers do not consider rare diseases also causes clinical studies which are not conducted in this area and diagnosis/treatment programs are not developed. A decision was taken to encourage research and create funds on rare diseases during a workshop held by the Turkish Ministry of Health in 2015, but it was not implemented (13).

Due to the lack of national plan or policies developed specifically for rare diseases in Turkey, the economic burden of rare diseases is often overlooked. For example, gluten-free foods, diapers, air humidifiers, breathing machines, wheelchairs, nebulizers, special shoes, ankle-foot orthosis and sunscreen, which individuals with rare diseases often have to use, can only be accessed through out-of-pocket payments. In addition, there are restrictions on the number of laboratory tests and physiotherapy sessions that are within the scope of reimbursement. Those above a certain number are covered through out-of-pocket payments. Also, doctors experienced in rare diseases generally serve in university hospitals or private hospitals. Although the exact number of doctors experienced in rare diseases is not known, it is known that there are difficulties in accessing the doctor. Therefore, patients have to pay a large amount of money to even just access to health care. However, even in health services covered by insurance, a very high number of “copayments” is required. As explained in the Social Security Institution Health Implementation Communiqué, 10–20% of copayments can be charged from the patients for the drugs covered by reimbursement in Turkey (14). Since a different policy has not been determined for rare diseases, patients may have to pay a contribution fee for high-priced orphan drugs. In a workshop carried out in Turkey on the problems experienced by individuals with rare diseases, it was stated that the biggest problem was the insurance coverage (15).

The literature review revealed no study about the out-of-pocket health expenses of patients with rare diseases; therefore, the economic burden created by these expenditures is unknown in Turkey. This study aims to determine the out-of-pocket health expenditures of Turkish households that include individuals with rare diseases.

## Materials and methods

To carry out the research, necessary permission was obtained from the Ondokuz Mayis University, Social and Human Sciences Research Ethics Committee. In this study, the out-of-pocket expenditures in rare diseases were examined and the examination was made from the perspective of the patient. All out-of-pocket expenditures of individuals with rare diseases were included in the study. Answering questions about spending habits and income is time consuming. However, individuals with rare diseases pay standard copayments because they routinely make these expenditures and their expenses are usually covered by GHI. Also, it was not difficult for them to provide their out-of-pocket expenditures as the average income of the majority of them is low. When there was an incomprehensible question, they contacted the principal investigator and asked for help.

### Research population

A registered data on the number of individuals with rare diseases in Turkey is not present and the only system in which these individuals are registered is the Rare Diseases Network. For this reason, the research population consisted of registered members of associations that are members of the Rare Diseases Network. The Rare Diseases Network is a patient association whose members are individuals with rare diseases. It serves to connect individuals with rare diseases, defend their rights and conduct awareness activities. Although approximately 4,000 individuals with rare diseases were registered in the Rare Disease Network, no sample population was selected from the research population because we aimed to reach the entire population. The study included individuals with only one rare disease and with no other family members with rare diseases in the household. When the individual with rare diseases was a child, was illiterate, or did not have the necessary information about the disease, necessary data were obtained from other family members (e.g., parents).

### Research questionnaire

A questionnaire was used as the data collection tool to determine the out-of-pocket healthcare expenditures related to the disease over the past year within the scope of the study. The questionnaire was developed based on national household health expenditures projects and expert opinions. The *Analysis of the Needs of Rare Disease Patients and Their Relatives, Common Mind Platform* project conducted by the Health Economics and Policy Association and The Scientific and Technological Research Council of Turkey (TUBITAK) provided information about the expenditures and problems of individuals with rare diseases (15). The questionnaire and methodology created through the *Turkey National Health Accounts and Household Health Expenditures 2002–2003* project conducted by the Ministry of Health and Hacettepe University (16) and the *Out-of-pocket Health Expenditures by Households in Turkey Survey* project by Hacettepe University in partnership with the World Bank (28) were taken as examples. In addition, the questionnaire was created in line with the opinions of health economists, medical doctors, individuals with rare diseases and their families. Medical doctors consulted during the survey process provided information about which health services individuals with rare diseases can use. The questionnaire was finalized by working with the members of the board of directors of the Rare Diseases Network consisting of specialists, patients and patient relatives. A pilot


$$\begin{array}{lll} The\;average\;expenditure & = & \frac{Total\;expenditure}{Total\;number\;of\;participants-Number\;of\;participants\;who\;did\;not\;respond}\\ Expenditure\;rate\;in\;annual\;household\;income & = & \frac{out\;of\;pocket\;expenditures-reimbursed\;amount}{annual\;household\;income} \end{array}$$

study was conducted with 20 households with the final form of the questionnaire and the questionnaire was made ready for the main study.

The first part of the questionnaire consisted of 14 questions and aimed to determine the demographic characteristics of the individuals with rare diseases. The second part included 11 questions about the rare disease and the last part including 30 questions was about the expenditures caused by the disease.

### Data collection

The online questionnaire form was available on Google Forms between May 1 and May 31, 2020. Necessary information to access the online survey was provided to the households by the Board of Directors of the Rare Diseases Network *via* e-mail and phone call.

### Data analysis

The total cost of the rare diseases consists of direct costs (drugs, medical devices and supplies, hospitalizations etc.) and indirect costs (loss of income due to the patients' or their relatives' absence from work or loss of productivity etc.). Direct costs include medical and non-medical costs. In this study, out-of-pocket expenditures like direct medical costs related to the disease such as drug copayments, private examination fees, and direct non-medical costs such as wigs and access to health services made by households were questioned. In addition to these expenditures, in case of presence of the income they lost due to the disease we tried to determine its amount. The total amount they spent and the income they lost could explain the economic burden of the household due to the disease.

After obtaining data on out-of-pocket healthcare expenditures, the necessary analysis was carried out using Microsoft Office Excel and Statistical Package for the Social Sciences (SPSS) V26. In addition to the general analysis including all participants, expenditures based on diseases were calculated. The costs calculated in Turkish Lira (Ł/TL) were converted to Euro (€) according to the average exchange rate for 2020.

After the responses were transferred to the Microsoft Office Excel software, the descriptive analyses were firstly made for the demographic and disease-related information of the participants. Then expenditures reported by the participants were calculated as minimum, maximum and average. Survey responses were analyzed separately as the general responses and the responses related to the disease.

In addition, the ratio of the average annual out-of-pocket health expenditure to the average household income was calculated. Accordingly, the number of households spending 10% and more than 100% of their average annual income was calculated.

After determining the out-of-pocket expenditures of individuals with rare diseases in the last one year, we examined whether there is a significant relationship between out-of-pocket health expenditures or not according to age, gender, income status and the patient's insurance type. Since the data did not show a normal distribution (*p* = 0.00), the Kruskal Wallis test was used to compare out-of-pocket health expenditures according to gender and insurance type during the analysis process; Spearman correlation coefficient test was used to compare the out-of-pocket health expenditures according to age and income. The significance level was taken as *p* < 0.05.

## Results

### Descriptive findings

A total of 472 households participated in the study. Thirty three questionnaires were not included in the analysis on the grounds that they were unqualified and contained incomplete information. After excluding the forms with missing information, a total of 439 forms were included in the analysis.

The first part of the research questionnaire contained demographic information about the individuals with rare diseases. Table 1 presents data on the demographic information of individuals with rare diseases. The average age of the patients was 11.7 (min:1, max: 70).

**Table 1:** Demographic characteristics of the participants.

**Age**	***n***	**%**
1–4	98	22.32
5–9	146	33.26
10–14	85	19.36
15–19	45	10.25
20–24	20	4.56
25–29	14	3.19
30–34	10	2.28
35–39	4	0.91
40–44	6	1.37
45–49	2	0.46
50–54	3	0.68
55–59	2	0.46
60–64	1	0.23
65+	1	0.23
Unspecified	2	0.46
Total	439	100
**Gender**		
Female	132	30.07
Male	307	69.93
Total	439	100
**Health insurance**		
General health insurance	405	92.26
Private health insurance	13	2.96
Both	21	4.78
Total	439	100

Table 2 presents information on the average annual income of the households, which was calculated as 41,513 TL (€5,251) (min: €1,012, max: €33,396).

**Table 2:** Average annual household income.

Annual income (€)^*^	***n***	**%**
0–1,265	2	0.46
1,265–2,530	46	10.48
2,530–3,795	92	20.96
3,795–5,060	111	25.28
5,060–6,325	63	14.35
6,325–7,590	39	8.88
7,590–8,855	42	9.57
8,855–10,120	14	3.19
10,120–11,385	9	2.05
11,385–12,650	9	2.05
12,650–13,915	5	1.14
13,915–15,180	1	0.23
15,180–16,445	3	0.68
16,445+	3	0.68

^*^The income of every family member who contributes to the household budget is included.

### Findings about the expenditures

After obtaining data on the treatment services, doctor's examinations, drugs, medical supplies and access to treatment, out-of-pocket healthcare expenditures regarding the disease were determined. The analysis included 439 households, and the expenditures were grouped: it was determined that special nutrition expenditures were the largest and emergency department expenditures were the smallest expenditure groups. Transportation, food and accommodation expenditures for access to healthcare services were ranked as the second, after special nutrition expenditures (Table 3).

**Table 3:** Out-of-pocket healthcare expenditures over the past year.

**Spending item**	**Total expenditure (Ł)**	**Average expenditure (Ł)**	Total expenditure (€)^*^	Average expenditure (€)^*^	**Percentage in total expenditure**
Special nutrition	1,251,866.00	3,320.60	158,361.05	420.06	%26.27
Drugs	568,090.00	1,623.11	71,863.39	205.32	%11.92
Laboratory and imaging tests	298,240.00	884.85	37,727.36	111.93	%6.26
Medical and non-medical devices and supplies	705,715.00	1,741.89	89.272,95	220.35	%14.81
Traditional/complementary medicine	219,715.00	507.42	27,793.95	64.19	%4.61
Emergency	19,905.00	51.57	2,517.98	6.52	%0.42
Hospitalization	274,190.00	708.50	34,685.04	89.63	%5.75
Transportation-food-accommodation	809,620.00	2,054.87	102,416.93	259.94	%16.99
Patient care^**^	380,000.00	865.60	48,070.00	109.50	%7.97
Specialist physician visit	238,557.00	596.39	30,177.46	75.44	%5.01
**Total**	**4,765,898.00**	**12,354.82**	**602,886.10**	**1,562.88**	**%100**

^*^1 TL = € 0.13.

^**^Patient care: includes expenditure on individuals recruited for the day care of individuals with rare diseases.

When a household member quits his or her job to care for the patient, a loss of income occurs. Adding the total loss of income to the total out-of-pocket healthcare expenditures reveals the cost of the disease. When data for all the participants was evaluated, the average cost of rare diseases was found as Ł22,743 (€2,877).

When the out-of-pocket healthcare expenditures of the participants were evaluated, 41% of them allocated more than 10% of annual household income to expenses related to the disease. A more important finding is that almost 7.52% of the respondents allocated more than all of their annual household income to disease-related expenditures.

In addition to the general analyses involving all participants, out-of-pocket healthcare expenditures based on diseases were also analyzed. This analysis included the rare diseases that more than 10 people had. Accordingly, the lowest and the highest amounts of money were spent on albinism and ectopia vesicae diseases, respectively. Besides, the average out-of-pocket healthcare expenditures for Duchenne Muscular Dystrophy (DMD), spinal muscular atrophy (SMA), cystinosis and cystic fibrosis diseases were close to each other (Table 4).

**Table 4:** Out-of-pocket healthcare expenditures based on disease.

**Disease**	**Total expenditure (Ł)**	**Average expenditure (Ł)**	**Total expenditure (€)**	**Average expenditure (€)**
Albinism	116,270.00	5,310.14	14,708.16	671.73
DMD	1,615,205.00	14,187.78	204,323.43	1,794.68
Ectopia Vesicae	169,190.00	15,348.94	21,402.54	1,941.64
Phenylketonuria	839,550.00	10,361.59	106,203.08	1,310.74
SMA	217,600.00	14,635.73	27,526.40	1,851.42
Cystinosis	290,664.00	14,510.24	36,769.00	1,835.55
Cystic fibrosis	788,413.00	13,882.11	99,734.24	1,756.09
All remaining diseases	729,006.00	11,045.55	92,219.26	1,397.26

Another topic we examined in our study was the presence of a statistically significant relationship between the characteristics and expenditures of the individuals with the disease. Table 5 presents the results of the analysis of determining whether there is a significant relationship between out-of-pocket health expenditures and age, gender, income status and insurance type. No significant relationship was found between gender (*p* = 0.098), age (*p* = 0.196) and the patient's insurance status (*p* = 0.246) and out-of-pocket health expenditures as a result of the analysis. However, a significant relationship was found between income status (*p* = 0.012) and out-of-pocket health expenditures.

**Table 5:** The relationship between age, gender, income status and insurance type and out-of-pocket health expenditures.

	***p***
Age	0,196
Gender	0,098
Income status	0,012
Insurance type	0,246

## Discussion

Compared to other diseases, rare diseases demonstrate very different characteristics in diagnosis and treatment stages and other disease-related processes. However, due to their low prevalence rare diseases are often overlooked and the policies are inadequate. For this reason, it is of great importance to determine the economic burden on the individuals living with rare diseases and their families as well as to develop necessary policies. This study was carried out for this specific purpose and out-of-pocket healthcare expenditure of individuals with rare diseases and their families were analyzed.

According to the analyses, the average age of the participants was quite low. Considering that rare diseases mostly affect children, it is an expected result. The average age of the patients was 11.7. 92% of participants were covered by general health insurance, almost 3% were covered by private health insurance and 5% had both types of insurance. Although all participants were covered by insurance, the high out-of-pocket healthcare expenditures indicate that there are unmet needs in rare diseases.

On the other hand, expenditure groups from highest to lowest were as follows: special nutrition expenditures (26.27%), transportation/accommodation/food expenditures (16.99%), medical and non-medical devices and supplies expenditures (14.81%), drugs expenditures (11.92%), patient care expenditures (7.97%), laboratory and imaging tests expenditures (6.26%), hospitalization expenditures (5.75%), specialist visits expenditures (5.01%), traditional and complementary medicine expenditures (4.61%) and emergency department services expenditures (0.42%). The fact that special nutrition expenditures account for a quarter of all expenditures shows the magnitude of the money spent on diet in these diseases and that there is no improvement in this field since there is no awareness in Turkey about this issue. As mentioned in the Introduction part, in Turkey, special nutrition expenditures are not covered by insurance and patients have to purchase special nutrition. According to a 2019 study on people with rare diseases, it was determined that 28% of the difficulties encountered in the treatment process in rare diseases were caused by management/system and policy, 23% by access difficulties and 19% by finance. These challenges are the lack of coverage of treatment services and drugs, the low coverage of medical devices, the expensiveness of low protein products, the lack of reimbursement due to the high costs of genetic therapies to correct the mutations seen in rare diseases and the failure to cover certain disease-specific nutrition (15). The researchers found that many of the difficulties experienced in management of rare diseases are due to economic problems related to treatment and medication, special nutrition and the medical supplies needed. Similarly, in the study conducted by Pak and Ince (17), the biggest problems of individuals with rare diseases were the necessity to go to developed cities to receive health services and the high drug costs in Turkey.

In a study conducted with 904 participants in China in 2014, total health expenditures and out-of-pocket expenditures in rare diseases were examined by Xin et al. (18) and they used the same survey method with this study. As a result of the study, the median total and out-of-pocket health expenditures were Renminbi (RMB) 20,000 Yuan and RMB 17,000 Yuan, with average RMB 58,120.3 Yuan and RMB 29,918.1 Yuan. In addition, a positive and significant relationship was found between out-of-pocket health expenditures and age, income status, type of insurance and hospitalization. Similarly, although there was a significant relationship between income status and out-of-pocket health expenditures in this study, no significant relationship was found between insurance status and age and out-of-pocket health expenditures. In the study, it was stated that critical illness insurance did not reduce out-of-pocket expenditures in rare diseases because the insurance coverage was very narrow and the reimbursement rate was low in China (18). In another study on critical illness insurance in China, it was stated that indirect costs such as transportation costs, private nutrition and wages lost due to illness are not covered by insurance, and that catastrophic health expenditure is inevitable in patients requiring high medical expenses (19). Similarly, in Turkey, costs such as transportation, special nutrition and loss of workforce are not covered by insurance, and rare disease holders with high medical expenses have to make out-of-pocket expenses as a special policy is not implemented for rare diseases.

In a study by Yang et al. (20), the economic burden of rare diseases in the United States in 2019 was calculated and the total economic burden was found to be 997 billion dollars. Of the total economic burden, 45% includes direct medical costs, 44% indirect costs, 7% non-medical costs and 4% of the costs were not covered by insurance. When indirect costs are examined; absenteeism costs ($149 billion), the cost of presenteeism ($138 billion) and losses from early retirement ($136 billion) are major costs for individuals and households with rare diseases. Uninsured and non-medical costs ($111 billion) include costs related with special equipment at home or in a personal/family vehicle (e.g., wheelchair), costs of transportation, required home renovations (e.g., ramp), day care, experimental, alternative or traditional treatments, and over-the-counter drugs. As stated in the study conducted by Yang et al. (20), the insurance does not cover medical and non-medical devices, caregiver, transportation, alternative medicine costs and costs of some drugs used in rare diseases in the United States of America (USA), as in Turkey.

Among the studies on the subject, the study by Lopez-Bastida et al. (21) shows analysis of quality of life and annual expenditures of individuals with rare diseases and their caregivers for specific countries. In the study, the difference of the items spent according to the type of disease and the costs varying in different countries were noted. This is due to the difference in the use of medication and healthcare under approval and reimbursement coverage in each country. Similarly, the study conducted by Gong et al. (22) examined the availability and affordability of orphan drugs in China. The average cost of 23 orphan drugs analyzed in the study was found to be $4,843.5 which is equal to the 505.6-day net income of a middle-income individual. The results show that availability of orphan drugs is low (20.8%) in China and orphan drugs are unaffordable for most patients.

It is observed that the economic cost of the disease was above 10% of the average annual income of 69.93% of the participants and above the average annual income of 25.97% of the participants. One of the important findings is that there is a significant relationship between income status and out-of-pocket health expenditures.

Considering the ratio of participants' out-of-pocket healthcare expenditures to their average annual income, we found that they allocate 30% of their average annual income to expenditures. We observed that 7.52% of the participants allocated more than all of their average annual income and 41% more than 10% of their average annual income to out-of-pocket healthcare expenses. It is understood that households experiencing this situation live under financial support provided by non-governmental organizations, municipalities, patient associations or by friends, relatives, neighbors or with bank loans. High out-of-pocket expenditures can lead to catastrophic expenditures. The catastrophic effect occurs when health expenditures exceed a certain threshold and generally creates an impoverishing effect on households. Although there are two different definitions of threshold in the literature, expenditures above 10% of total household income and 40% of non-food expenditures (payment capacity) are considered catastrophic (23). An evaluation in accordance with this explanation would mean that almost 70% of participants with rare diseases are exposed to catastrophic health expenditures. In the study conducted by Wang et al. (24) in China between 2014 and 2015, out-of-pocket expenditures for phenylketonuria were analyzed and it was found that the economic burden caused by phenylketonuria constituted 75% of the total annual family income and 94% of households made catastrophic health expenditures. It was seen that 58.3% of the total economic burden consisted of special nutrition (phenylalanine-free formulas) expenditures, 9.9% of low-protein rice and flour expenditures and 3.9% of accommodation and transportation expenditures for access to treatment. The findings of the study conducted by Wang et al. (24) are similar to the findings of our study. Special nutrition, access to treatment and medical and non-medical device expenditures in rare diseases are not reimbursed in most countries as in Turkey, and patients have to pay out of pocket. However, this leads to catastrophic health expenditures for most households.

When expenditures for the rare diseases that more than 10 people had were specifically analyzed, it was found that the highest amounts of money were spent for the ectopia vesicae disease. In addition, average expenditures for DMD, cystic fibrosis, cystinosis and SMA diseases were similar. The disease with the lowest expenditure was determined as albinism. Also, it was seen that the topic of expenditure varied depending on the disease. Although similar amounts of money were spent, expenditure groups from highest to lowest were found to be as follows: medical and non-medical devices and supplies in albinism (31%) and SMA (41%), special nutrition in DMD (20%) and phenylketonuria (72%), and transportation/accommodation/food expenses in ectopia vesicae (47%), cystic fibrosis (24%) and cystinosis (32%). Similar to the results of this study, it was observed that the highest expenditure group (58.3%) in phenylketonuria disease was special nutrition in the study conducted by Wang et al. (24).

Since no study is present about the economic burden of rare diseases or out-of-pocket expenditures in rare disease in Turkey, it is not possible to compare the changes over time. As stated in the study by Ince and Pak Güre (25), rare diseases policies in Turkey are quite limited and there is no national plan developed and implemented for rare diseases yet. While more than 100 orphan drugs are covered by reimbursement in many countries such as the USA, Netherlands, Germany and France (25), only 34 orphan drugs are covered in Turkey (26). In addition, while there are only 6 different screening programs for rare diseases in Turkey, there are 28 different programs in Poland, 20 in the Netherlands, 15 in Germany and 5 in France. Considering the existence of activities initiated in the USA and the European Union in 1980, there are many steps that Turkey needs to take in developing a health policy in the field of rare diseases (1). Out-of-pocket expenditures must be reduced by development of policies to be applied in the field of rare diseases and by expanding the insurance coverage according to the needs of rare disease holders.

In the study by Ince and Pak Güre (25), Multi-Objective Optimization based on Ratio Analysis (MOORA), Complex Proportional Assessment (COPRAS) and Technique for Order Preference by Similarity to Ideal Solution (TOPSIS) techniques were used to evaluate the policy performances of 18 OECD countries on rare diseases through health policy indicators. As a result of the study, Germany was ranked the first for all the methods used while France, Netherlands, United Kingdom and Italy formed the subsequent ranking. Turkey is at the bottom of the list, behind Latvia and Greece. As can be understood from the studies carried out, government policies on rare diseases in Turkey are quite inadequate when compared to other countries. As seen in this study, people with rare diseases require different needs, such as special nutrients, medical devices and supplies and orphan drugs compared to common diseases. The inadequacy of policies in the field of rare diseases in Turkey compared with other countries confirm the results of this study. As seen in this study, 7.52% of the participants allocate their entire income to out-of-pocket expenses. This shows that policies in the field of rare diseases in Turkey are insufficient.

This study is crucial because it provides a clear picture of the economic difficulties experienced by individuals with rare diseases. According to the results of the study, the out-of-pocket expenditures of individuals with rare diseases to special nutrition, transportation, food, accommodation, drugs, medical and non-medical devices and supplies constitute a high economic burden. In Turkey, transportation and accommodation expenditures made by patients in order to reach the physicians who are interested and experienced or specialized in the field of rare diseases are not taken into account by any legislation. The fact that experienced doctors in this field generally work in private and university hospitals that mainly locate in center causes patients living in rural areas to regularly spend money on transportation and accommodation. In addition, it is a critical need to make legal arrangements regarding special nutritional products, drugs and medical and non-medical devices used by rare disease holders. Inclusion of special nutritional products and medical and non-medical devices used in rare diseases within the scope of reimbursement and the development of orphan drug legislation are the first actions to be taken by policy makers. In addition, the Rare Diseases National Action Plan should be published and implemented as soon as possible. This is a step that needs to be taken in Turkey so that rare diseases are treated distinctively, not equally with the common diseases.

## Data availability statement

The data analyzed in this study is subject to the following licenses/restrictions: The dataset is depending on a survey of individuals. Publishing the dataset may result in loss of anonymity. Requests to access these datasets should be directed to hello@econix.net.

## Author contributions

Data collection, literature review, and writing: SÖ. Data analysis: MK. Theory, interpretation, revision, and writing: GO and GK. All authors contributed to the article and approved the submitted version.
