# Supplementary material for: Out-of-pocket healthcare expenditures of Turkish households living with rare diseases
Source: Front Public Health. 2023 Mar 3;11:1051851. doi: 10.3389/fpubh.2023.1051851 (PMC10020592; doi:10.3389/fpubh.2023.1051851)
Supplement: Supplementary file 1 [file Data_Sheet_1.DOCX]

**HEALTH EXPENDITURES MADE BY HOUSEHOLDS
LIVING WITH RARE DISEASES**

Dear participant;

Out-of-pocket expenses are expenses you make entirely out of your own pocket, not covered by your insurance. Participation in the study is on a voluntary basis. The data to be collected from you will only be used in educational and scientific activities within the scope of the research, this information will not be shared with any other person or institution for any other purpose, and your privacy will be strictly ensured. The accuracy of your answers to the questions in the questionnaire is very important in terms of the quality of the research. Therefore, we ask you to answer the questions in the survey correctly. Thank you for your help.

** The questionnaire can be filled by the patient or the patient's relative.

Gülpembe Oğuzhan

Department of Health Management

[gulpembe.oguzhan@omu.edu.tr](mailto:gulpembe.oguzhan@omu.edu.tr)

+905333993431

**SECTION 1: PATIENT'S DEMOGRAPHIC INFORMATION**

|  | I am ( ) Patient ( ) Patient’s relative…………………….….. | | | | | |
| --- | --- | --- | --- | --- | --- | --- |
|  | Patient's date of birth (Please write year):………….................. | | | | | |
|  | Patient's gender | ( ) Female | ( ) Male |  |  | |
|  | Patient's marital status | ( ) Single | ( ) Married | Other.................. | | |
|  | Patient's education status |  |  |  | |  |
|  | ( ) Not literate | ( ) Only write-read | ( ) Primary school | ( ) Middle School | |  |
|  | ( ) High School | ( ) University | ( ) Master |  | |  |
|  | Patient's working status | ( ) Working | ( ) Not working  **(Please proceed to question 11)** | ( ) Retired | |  |
|  | Patient's occupation |  |  |  | |  |
|  | ( ) Self-employment | ( ) Worker | ( ) Officer |  | |  |
|  | ( ) Student |  | ( ) Other:…………………………… | | | |
|  | Patient's annual income (you can give an approximate answer by considering the year 2019) ........................................................... | | | | | |
|  | Are you unable to work because of your illness? ( ) Yes ( ) No **(Please proceed to question 11)** | | | | | |
|  | I ( ) have ( ) have not lost income. If you have a loss of income, please specify in Turkish lira.…………………………..TL | | | | | |
|  | Annual income of the household where the patient lives (You can give an approximate answer by considering the year 2019, write by adding up with the patient's).........................TL | | | | | |
|  | Patient's health insurance type ( ) General Health Insurance ( ) Private Health Insurance  ( ) Both | | | | | |

**SECTION 2: DISEASE QUESTIONS (Please focus on your rare disease in this section)**

|  | What is the diagnosis of your disease?......................................................................................... | |
| --- | --- | --- |
|  | Which branches have you visited in the last 1 year regarding your illness?  Branch 1: …….................................................……...(……………times)  Branch 2: ……........................................................... (……………times)  Branch 3: ……........................................................... (……………times)  Branch 4: ……........................................................... (……………times)  Branch 5: ……........................................................... (……………times)  ………………………………………………………………..  ……………………………………………………………….. | |
|  | In the last 1 year, have you had to go out of your village/town/province to receive treatment for your illness? ( ) Yes ( ) No (Please proceed to question 6) | |
|  | What is the distance you travel to get treatment? (Please specify in km)......................... | |
|  | Did you go to a private hospital compulsorily (for reasons such as not being able to make an appointment / finding a specialist doctor, etc.) to receive treatment for your disease? ( ) Yes  ( ) No | |
|  | What medications do you use for your rare disease?  ....................................................................................................................................................  ………………………………………………………………………………………………… | |
|  | What are the medical supplies, tests and health services you use outside the hospital for this disease?  ....................................................................................................................................................  ………………………………………………………………………………………………… | |
|  | Have you not been able to take medicine for your rare disease in the last 1 year because of its price, or have you taken less than necessary? ( ) Yes ( ) No | |
|  | Have you been unable or under-received medical equipment, medical tests, or health care for your rare disease in the last 1 year because of the price? ( ) Yes ( ) No | |
|  | Do you have a chronic disease other than your rare disease? | |
|  | ( ) Yes | ( ) No (Please proceed to Section 3) |
|  | What is/are this chronic disease? ...................................................................................................................... | |

**SECTION 3: EXPENDITURES ON RARE DISEASE TREATMENT**

**IMPORTANT EXPLANATION: Answer this section considering only the payments you have made for your rare disease. Answer for the last 1 year. Think backwards. You can calculate your total annual expenditure based on your monthly or daily expenditures. You can reach the annual total by considering the procedures and examinations every time you go.**

|  | Is there any special nutritional food or diet that you started to consume due to your rare disease?  If yes, please indicate your total annual expenditure. | ( ) Yes--->  ( ) No | ( ) No payment  ( ) Paid ……………….TL  ( ) Don’t know |
| --- | --- | --- | --- |
|  | Do you take medicine for rare disease?  If yes, please indicate your total annual expenditure. | ( ) Yes--->  ( ) No | ( ) No payment  ( ) Paid ……………….TL  ( ) Don’t know |
|  | Were laboratory tests performed for the treatment?  If yes, please indicate your total annual expenditure. | ( ) Yes--->  ( ) No | ( ) No payment  ( ) Paid ……………….TL  ( ) Don’t know |
|  | Were any procedures such as MRI, x-ray, ultrasound, angiography, endoscopy, tomography performed for the treatment? If yes, please indicate your total annual expenditure. | ( ) Yes--->  ( ) No | ( ) No payment  ( ) Paid ……………….TL  ( ) Don’t know |
|  | Were medical devices and equipment such as walking sticks, wheelchairs, glasses/lenses, hearing aids, special beds or prostheses used in the treatment? If yes, please indicate your total annual expenditure. | ( ) Yes--->  ( ) No | ( ) No payment  ( ) Paid ……………….TL  ( ) Don’t know |
|  | Were medical materials such as bandages, cotton, elastic bandages, urine bags, injectors used in the treatment?  If yes, please indicate your total annual expenditure. | ( ) Yes--->  ( ) No | ( ) No payment  ( ) Paid ……………….TL  ( ) Don’t know |
|  | Was anything other than the ones listed in the above questions (such as a wig) taken for the treatment?  If yes, please indicate your total annual expenditure. | ( ) Yes--->  ( ) No | ( ) No payment  ( ) Paid ……………….TL  ( ) Don’t know |
|  | Have you applied to traditional treatment methods and/or complementary medicine for the treatment of the disease? (Acupuncture, hypnosis, leech application, etc.) | ( ) Yes  ( ) No **(Please proceed to question 10)** | |
|  | Did you pay directly for this healthcare service?  (Write the cash value of non-monetary payments) If yes, please indicate your total annual expenditure. | ( ) No payment  ( ) Paid ……………….TL  ( ) Don’t know | |
|  | Have you ever been to the emergency in connection with a rare disease? | ( ) Yes  ( ) No **(Please proceed to question 12)** | |
|  | Did you make any payment for the emergency service received? If yes, please indicate your total annual expenditure. | ( ) Yes--->  ( ) No | ( ) No payment  ( ) Paid ……………….TL  ( ) Don’t know |
|  | Have you been hospitalized in connection with the rare disease? | ( ) Yes  ( ) No **(Please proceed to question 14)** | |
|  | Have you made any payment for your hospital stay? If yes, please indicate your total annual expenditure. | ( ) Yes--->  ( ) No | ( ) No payment  ( ) Paid ……………….TL  ( ) Don’t know |
|  | Have you paid for transportation, food and accommodation for the treatment of the rare disease?  (Please indicate the total costs of the patient and attendant.) If yes, please indicate your total annual expenditure. | ( ) Yes--->  ( ) No | Transportation ………………………TL  Food ………………..……..TL  Accommodation ………………….TL |
|  | Do you need someone to care for the patient due to the disease?  If yes, please indicate your total annual expenditure. | ( ) Yes--->  ( ) No | ( ) No payment  ( ) Paid ……………….TL  ( ) Don’t know |
|  | If the person caring for the patient is from the household, did he/she have to quit his/her job?  If yes, please indicate your total annual income loss. | ( ) Yes--->  ( ) No | Please specify in Turkish Lira ……….………………………TL |
|  | Have you made a payment for a specialist physician examination for the follow-up of the disease? If yes, please indicate your total annual expenditure. | ( ) Yes--->  ( ) No | ( ) No payment  ( ) Paid ……………….TL  ( ) Don’t know |
|  | Has any institution reimbursed you for all the expenses you have made so far for the treatment of the disease? If yes, please indicate your total annual expenditure. | ( ) Yes --->  ( ) No  ( ) Don’t know | Please specify in Turkish Lira ……….………………………TL |

You can write anything you want to add about the subject here. …………………………………………………………………….
